# A Case of Twins with Malpresentation

**Published:** 1855-10

**Authors:** 


THE NORTH-WESTERN

MEDICAL AND SURGICAL JOURNAL.

NEW SERIES.

VOL. IV. OCTOBER, 1855. NO. 10.

### ORIGINAL COMMUNICATIONS.

ARTICLE I.—*A Case of Twins with Malpresentation.*

JULY 27th, 1855.—Called at 4 o'clock, A. M., to Mrs. Daily, a native of Ireland, aged 42 years—in her tenth confinement. Learned from the attending midwife that she had been in active labor since six o'clock of the former evening—a duration of ten hours; the pains still continued strong and severe. On examination, per vaginum, found the os uteri fully dilated, the walls of the vagina moist and yielding, but the presentation so complicated and novel, that I felt reluctant to hazard any diagnosis or further procedure without counsel, I therefore sent for Prof. Freer, who, on making an examination pronounced the presentation to be one in which two fœtal heads occupied the right and left illiac fossæ respectively, with the right arm and prolapsed funis of the right child presenting intermediately. The accuracy of this diagnosis was fully confirmed by subsequent manipulations.

Our first aim was to correct the malposition of the head of the right child, and to place it in the most favorable relation to the diameters of the pelvis. The arm and funis were gently pushed up and retained above the brim, until the malposition of the head, by means of the vectis and external abdominal manipulation, was corrected: but the tendency of the head to recede was so great that it was impossible to prevent its return into the iliac fossæ. The retention of the head at the brim for a sufficient length of time to admit of the application of the forceps was impracticable. Finally, version was agreed upon. A succession of attempts was made by both without success. The powerful contractions of the uterus which supervened on introducing the hand, the seeming multiplicity of extremities and the anomalous tortuosities of the cord, all tended to frustrate the explorations for the feet.

Prof. Blaney, was now sent for to share the fatigue and responsibility. In the meantime Prof. Freer, with praiseworthy perseverance made another attempt by which he succeeded in securing one extremity of the right child, at the flexor of the knee. But it was not until firm and long continued traction had been made that the arm and head receded and the foot was brought down. The first child was then delivered as in breech presentations,—the child still-born.

After the birth of the first child a sufficient amount of hemorrhage occurred to warrant the immediate introduction of the hand into the uterus, which detected an hour glass contraction, including the placenta, and second child above the point of contraction. The feet were immediately seized—version effected, and to avoid detail, delivery completed in a very short space of time. The child alive—but requiring artificial respiration for its resuscitation.

The placenta was removed without any unusual difficulty. It was a single placenta of large size, furnished with two sets of membranes. The size of the uterus was enormous and the abdomen pendulous. During the whole period of labor the patient retained her strength, notwithstanding the severity of the manipulations, and the almost continuous succession of the pains.—An excellent example of Irish tenacity of life.

At half past twelve, P. M., left her, ordered ergot lest hemorrhage should occur, the uterus not having contracted perfectly.

In the after treatment of this case there have been no symptoms differing from those observed in convalescence from natural labor.

The novelty of this case may be thus summed up, as consisting in the presentation of two fœtal heads—an arm and a prolapsed funis, in the singleness of the placenta, and in the unusual contortions and intricate windings of the cord. To the above summary may be added the complications of an hour glass contraction and uterine hemorrhage.

